# Targeting Sonic Hedgehog Signaling by Compounds and Derivatives from Natural Products

**DOI:** 10.1155/2013/748587

**Published:** 2013-05-20

**Authors:** Yu-Chuen Huang, K. S. Clifford Chao, Hui-Fen Liao, Yu-Jen Chen

**Affiliations:** ^1^School of Chinese Medicine, China Medical University, Taichung 404, Taiwan; ^2^Graduate Institute of Biostatistics, China Medical University, Taichung 404, Taiwan; ^3^Department of Medical Research, China Medical University Hospital, Taichung 404, Taiwan; ^4^Department of Radiation Oncology, Columbia University, New York, NY 10032, USA; ^5^Department of Biochemical Science and Technology, National Chiayi University, Chiayi 600, Taiwan; ^6^Institute of Traditional Medicine, National Yang-Ming University, Taipei 112, Taiwan; ^7^Department of Radiation Oncology and Department of Medical Research, Mackay Memorial Hospital, 92 Chung-Shan North Road, Section 2, Taipei 104, Taiwan

## Abstract

Cancer stem cells (CSCs) are a major cause of cancer treatment failure, relapse, and drug resistance and are known to be responsible for cancer cell invasion and metastasis. The Sonic hedgehog (Shh) signaling pathway is crucial to embryonic development. Intriguingly, the aberrant activation of the Shh pathway plays critical roles in developing CSCs and leads to angiogenesis, migration, invasion, and metastasis. Natural compounds and chemical structure modified derivatives from complementary and alternative medicine have received increasing attention as cancer chemopreventives, and their antitumor effects have been demonstrated both *in vitro* and *in vivo*. However, reports for their bioactivity against CSCs and specifically targeting Shh signaling remain limited. In this review, we summarize investigations of the compounds cyclopamine, curcumin, epigallocatechin-3-gallate, genistein, resveratrol, zerumbone, norcantharidin, and arsenic trioxide, with a focus on Shh signaling blockade. Given that Shh signaling antagonism has been clinically proven as effective strategy against CSCs, this review may be exploitable for development of novel anticancer agents from complementary and alternative medicine.

## 1. Introduction

 Cancer stem cells (CSCs) are a small minority of cancer cells that can proliferate extensively and form new tumors [1–4]. These properties of CSCs are thought to cause cancer treatment failure, relapse, and drug resistance [5]. As with normal stem cells, CSCs are self-renewing and can differentiate into phenotypically diverse tumor and nontumor cancer cells [2]. Several embryonic signaling pathways are known to be involved in normal stem cell maintenance, and these have recently been linked to carcinogenesis and tumor propagation. Indeed, signaling pathways that support dysregulated self-renewal and proliferation of CSCs may provide practical targets for preventing tumor regrowth and improving treatment outcomes. 

 Sonic hedgehog (Shh) signaling is critical to embryogenesis and is essential for the development of several tissue types and organs [6]. Aberrant activation of Shh signaling is central to CSC activities, which promotes tumor progression, angiogenesis, migration, invasion, and metastasis [7–9]. Dysregulation of Shh signaling has been involved with several malignancies, including basal cell carcinomas, medulloblastomas, leukemia, oral squamous cell carcinomas, and gastrointestinal, pancreas, lung, ovarian, breast, and prostate cancers [10–14]. Shh is a secreted glycoprotein that activates signaling by binding to the transmembrane receptor patched-1 (Ptch1). Subsequently, Ptch1-mediated inactivation of Smoothened (Smo) is reversed, allowing transduction of the Shh signal, which results in nuclear translocation of cytoplasmic transcription factors of the Gli family to modulate target gene expression [15]. 

**Figure 1:**
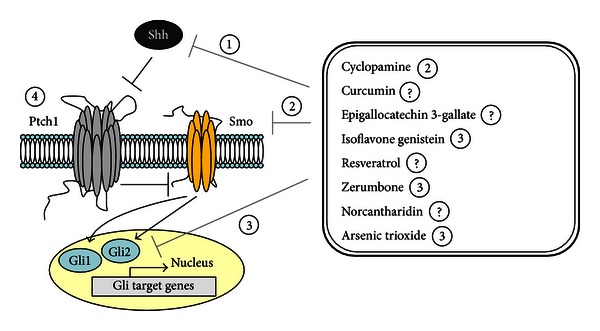
Targeting the Sonic hedgehog signaling pathway by compounds and derivatives from natural products. Circled numbers beside compound names indicate target Shh signaling proteins. Question marks indicate that the targeted protein is uncertain.

 Since CSCs are considered as a cause of cancer treatment failure, relapse, and drug resistance, therapeutic targeting of CSCs may overcome tumor resistance, reduce relapse, and improve patient survival. The small molecule Smo inhibitor vismodegib has been demonstrated effective in reducing the basal-cell carcinoma tumor burden. However, the adverse events caused discontinuation in over half of treated patients [16]. It implicates that Shh signaling blockade is an effective strategy and remains a room to develop effective agents with more favorable safety profile. Several naturally occurring dietary compounds have shown promise as Shh signaling inhibitors. In this paper, we review the current understanding of some natural compounds that have cancer treatment potential, with a focus on targeting the Shh signaling pathway (Figure 1). 

## 2. Compounds and Derivatives from Natural Products That Regulate Sonic Hedgehog Signaling Pathway

### 2.1. Cyclopamine

 Cyclopamine is a naturally occurring compound from the plant *Veratrum californicum*, commonly known as corn lily, and was the first phytochemical, which demonstrated Shh pathway inhibitory activity [17, 18], and was shown to block the activation of Smo [18]. Berman et al. reported that treatment of murine medulloblastoma cells with cyclopamine inhibited proliferation, induced neuronal differentiation, and depleted CSC populations [17]. It also caused regression of murine tumor allografts and induced rapid death of cells from freshly resected human medulloblastomas [17]. Cyclopamine may inhibit development and invasiveness of human hepatocellular carcinomas (HCCs) *in vitro* and *in vivo* by inhibiting the Shh pathway. Blockade of Shh signaling may be a potential target of new therapeutic strategy for HCC [19–22]. In addition, cyclopamine effectively targeted CSCs of pancreatic cancer, breast cancer, glioblastoma, and multiple myeloma [23, 24]. 

### 2.2. Curcumin

 Curcumin, the major extract of tumeric, is derived from the plant* Curcuma longa*, which is a common ingredient of mustard and curry [25, 26]. It has been widely used in traditional medicines and dietary supplements for centuries [27]. Curcumin has anti-inflammatory and antioxidant activities and has been studied as a preventive agent in several cancer models [25, 28]. After treating medulloblastoma cells with curcumin, Elamin et al. observed that curcumin caused inhibition of Shh signaling cell growth and induction of apoptotic cell death by downregulating proteins of the Shh pathway. In addition, curcumin enhanced the antitumor effects of cisplatin and *γ*-rays by targeting pathways that are crucial for tumor survival [29]. Moreover, curcumin inhibited prostate cancer cell growth through the Shh pathway and reduced or delayed prostate cancer growth in a transgenic adenocarcinoma mouse model [30].

### 2.3. Epigallocatechin-3-Gallate

 Green tea is one of the most widely consumed beverages in the world [31]. Epigallocatechin-3-gallate (EGCG) acts as a potent cancer-preventive agent through various mechanisms and is the most abundant polyphenolic catechin in green tea [32]. Specifically, Tang et al. reported inhibition of the Shh pathway, induction of apoptosis, and suppression of human chondrosarcoma cell proliferation by EGCG, suggesting that EGCG could be a new therapeutic agent for patients with chondrosarcoma [33]. Moreover, EGCG was shown to inhibit prostate cancer cell growth by suppressing Gli1 mRNA expression and downregulating Gli promoter activity [30]. In transgenic prostate adenocarcinoma mice, EGCG reduced or delayed prostate tumor growth [30]. A recent report showed that EGCG inhibited the components of Shh pathway and Gli transcriptional activity in pancreatic CSCs. It also inhibited self-renewal capacity of pancreatic CSCs, and these effects were enhanced in the presence of the flavonoid quercetin, which is present in fruits and vegetables such as onion, tea, apples, and berries [11]. These data suggest that EGCG either alone or in combination with quercetin could be used to prevent and treat pancreatic cancer [11].

### 2.4. Isoflavone Genistein

 Soybeans are an abundant source of isoflavones. Genistein is one of the most active soy isoflavones. Numerous studies report protective effects of soy foods against breast and prostate cancers [34–37]. Indeed, soy isoflavones, particularly genistein, exert potent antiproliferative effects on breast, prostate, colon, skin, gastric, and bladder cancers [38]. In transgenic prostate adenocarcinoma mice, genistein inhibited cancer cell growth through its actions on the Shh pathway and reduced or delayed growth of prostate tumors [30]. In a study by Zhang et al., genistein not only inhibited prostate cancer cell invasion [39] but also targeted prostate CSCs and suppressed tumorigenicity [40]. This anti-CSC effect was due to inhibition of the Shh protein Gli1 [40], suggesting that genistein may be a potent chemopreventive agent against prostate CSCs.

### 2.5. Resveratrol

 Resveratrol is a dietary polyphenol derived from plants such as grapes, berries, plums, peanuts, and *Polygonum cuspidatum* [41]. Resveratrol inhibited the proliferation of a wide variety of human cancer cells *in vitro* and slowed carcinogenesis in animal models [42, 43], suggesting therapeutic and cancer preventive effects [43]. Ślusarz et al. demonstrated involvement of Shh signaling in resveratrol-mediated inhibition of prostate cancer cell growth *in vitro *and *in vivo *[30]. Moreover, resveratrol inhibited both Shh signaling and Bcr-Abl expression in human chronic myeloid leukemia (CML) cells, indicating that resveratrol may have potential as a treatment for CML [10]. Recently, another report showed that resveratrol could effectively downregulate interleukin-6-stimulated Shh signaling in human acute myeloid leukemia [44]. 

### 2.6. Zerumbone

*Zingiber zerumbet* Smith is a perennial, tuberous root herb that grows mainly in southeast Asia [45]. The major extract zerumbone has been shown to increase apoptosis and inhibit cancer cell invasion and has demonstrated antitumor effects against leukemia and breast, lung, liver, and pancreatic cancers [46–49]. Hosoya et al. reported that zerumbone inhibited both Gli1- and Gli2-mediated transcription and repressed the transcription of other Shh signaling genes, including Ptch1 and BCL2, in HaCaT cells [50].

### 2.7. Norcantharidin

 The small-molecule norcantharidin (NCTD) is a demethylated synthetic analog of naturally occurring cantharidin from blister beetles (*Mylabris phalerata* Pall.). The effects of NCTD against diverse malignancies have been investigated. It has been shown capable of inducing cell anoikis and apoptosis [51], inhibiting invasion and angiogenesis [52], and suppressing metastasis [53]. Moreover, NCTD may overcome multidrug resistance by inhibiting Shh signaling and expression of downstream multidrug resistance (MDR1) P-glycoprotein in human breast cancer cells [13]. 

### 2.8. Arsenic Trioxide

 Arsenic trioxide (As_2_O_3_) has been used therapeutically in traditional Chinese medicine for a long time and has been shown as a highly effective treatment for relapsed acute promyelocytic leukemia [54]. In addition, arsenic trioxide can inhibit Shh signaling at the Gli protein level, although the exact mechanism remains controversial. Kim et al. demonstrated that arsenic trioxide antagonizes Shh signaling primarily through interference with Gli2 [55]. Moreover, Beauchamp and colleagues provide evidence that arsenic trioxide could inhibit the growth of Ewing sarcoma and medulloblastoma cells by targeting Gli1 [56]. These data suggest that arsenic trioxide could be used as a therapeutic agent in malignant diseases associated with Shh pathway activation.

### 2.9. Clinical Implications of Natural Compounds and Derivatives

 Some natural compounds and their derivatives have potent antitumor effects, offering clues to the design of targeted therapeutic anticancer agents. In addition to affecting cancer related signaling pathways, natural compounds may synergize with chemotherapy and radiotherapy as antitumor agents. For example, Choi et al. showed pretreatment with zerumbone before radiation induced radiosensitization in human lung adenocarcinoma cells and in a xenograft mice model [57]. Curcumin, genistein, and EGCG have potential in the treatment of prostate cancer [30, 39, 40]. Moreover, resveratrol and NCTD are potential treatments for acute myeloid leukemia and breast cancer, respectively [13, 44]. Hence, further development of molecular tests that assess tumor tissue and DNA from patients will aid therapeutic decisions to use natural compounds. In addition, molecular markers of Shh signaling may facilitate the development of personalized cancer treatments.

## 3. Conclusions

 In conclusion, the antitumor effects of naturally occurring compounds that target Shh signaling indicate the importance of this pathway to cancer cell invasion and metastasis. Hence, these natural compounds and related derivatives may be used as primary treatment or as adjunctive agents for combinatory treatment to improve therapeutic index against cancer. Although this review focuses only on natural compounds and related derivatives that target the Shh signaling pathway, the data summarized herein indicate that complementary and alternative medicine may comprise a multitude of compounds with potential to prevent cancer and its metastasis by targeting various signaling pathways. Further *in vitro* and *in vivo* studies, as well as clinical trials, are warranted to investigate the therapeutic potential of natural compounds against CSCs.
